# Increasing risk of cataract in HCV patients receiving anti-HCV therapy: A nationwide cohort study

**DOI:** 10.1371/journal.pone.0173125

**Published:** 2017-03-06

**Authors:** Shih-Yi Lin, Cheng-Li Lin, Shu-Woei Ju, I-Kuan Wang, Cheng-Chieh Lin, Chih-Hsueh Lin, Wu-Huei Hsu, Ji-An Liang

**Affiliations:** 1 Graduate Institute of Clinical Medical Science, College of Medicine, China Medical University, Taichung, Taiwan; 2 Division of Nephrology and Kidney Institute, China Medical University Hospital, Taichung, Taiwan; 3 Management Office for Health Data, China Medical University Hospital, Taichung, Taiwan; 4 College of Medicine, China Medical University, Taichung, Taiwan; 5 Department of Family Medicine, China Medical University Hospital, Taichung, Taiwan; 6 Division of Pulmonary and Critical Care Medicine, China Medical University Hospital and China Medical University, Taichung, Taiwan; 7 Department of Radiation Oncology, China Medical University Hospital, Taichung, Taiwan; Chiba University, Graduate School of Medicine, JAPAN

## Abstract

**Purpose:**

Hepatitis C virus (HCV) infection is associated with increased systemic oxidative stress, which leads to cardiovascular events, diabetes, and chronic kidney disease. Similarly, cataract is also associated with increased oxidative stress. The association between HCV infection and increased risk of cataract remains unclear.

**Methods:**

A total of 11,652 HCV-infected patients and 46,608 age- and sex-matched non-HCV infected patients were identified during 2003–2011. All patient data were tracked until a diagnosis of cataract, death, or the end of 2011. Cumulative incidences and hazard ratios (HRs) were calculated.

**Results:**

The mean follow-up durations were 5.29 and 5.86 years for the HCV and non-HCV cohorts, respectively. The overall incidence density rate for cataract was 1.36 times higher in the HCV cohort than in the non-HCV cohort (1.86 and 1.37 per 100 person-y, respectively). After adjusting for age, sex, comorbidities of diabetes, hypertension, hyperlipidemia, asthma, chronic obstructive pulmonary disease, coronary artery disease, and anxiety, patients with HCV infection had an increased risk of cataract compared with those without HCV infection [adjusted HR = 1.23, 95% confidence interval (CI) = 1.14–1.32]. HCV-infected patients receiving interferon–ribavirin therapy had a 1.83 times higher (95% CI = 1.40–2.38) risk of cataract than non-HCV infected patients did.

**Conclusion:**

HCV infection, even without the complication of cirrhosis, is associated with an increased risk of cataract, and this risk is higher in HCV-infected patients undergoing interferon–ribavirin therapy.

## Introduction

Cataract, which has an estimated prevalence of 33% in the general population, is the second leading cause of visual impairment and vision loss worldwide [1]. Cataract is associated with increasing incidences of falls and traffic accidents and impair self-care ability and quality of life [2]. Increasing associated medical expenditures makes cataract being a major public health concern [3]. Aging is the major etiology of cataract. Meanwhile, cataract can also be hereditary or result from other eye conditions, trauma, previous eye surgery, long-term steroid use, and several medical conditions such as diabetes [4–6]. The pathogenesis of cataract is complex, and includes photochemical generation of reactive oxygen species, oxidative stress [7], DNA damage, polyol formation [8], osmotic stress, as well as protein misfolding and aggregation [9]. Several experimental animal studies have shown that topical antioxidants such as caffeine, ascorbate, and vitamin Effectively prevent cataract formation and that oxidative stress play a central role in cataract pathogenesis [10 11].

Hepatitis C virus (HCV) infection has an estimated global prevalence of 2.2% and is also a major health concern worldwide [12]. HCV leads to liver cirrhosis, hepatic decompensation, and hepatocellular carcinoma. HCV infection has also various extrahepatic presentations, including renal dysfunction, cardiovascular events, and metabolic syndromes; these extrahepatic presentations are believed to be related with HCV-induced systemic oxidative stress and hyperinsulinemia [13].

Although cataract is also a manifestation of oxidative stress and has long-term effects involving visual impairment, the association between cataract risk and HCV infection has seldom been investigated in detail. Yoshida et al have reported that patients with age-related cataract had significantly higher seropositivity for HCV than an age-matched general population [14]. In this study, we investigated this association by using the National Health Insurance (NHI) database of Taiwan, which is a nationwide longitudinal database with medical claims of 23 million people. Additionally, this study examined whether HCV infection is a risk factor for cataract.

## Methods

### Data source

In Taiwan, National Health Insurance (NHI) is a single-payer program that has been launched since 1995, covering 98% of the population [15]. National Health Insurance handled a research database (NHIRD), which encompassed patient demographics, diagnosis of disease, mediations, and procedures performed in the hospital and in outpatient claims. Several datasets were extracted from the complete NHIRD for researches purposes. This population-based retrospective cohort study was conducted by analyzing one of the extracted set of NHIRD, the Longitudinal Health Insurance Database 2000 (LHID2000) of the NHI program. The details of the NHI program and LHID2000 have been well-addressed previously [16, 17].

### Ethics statement

The NHIRD encrypts patient personal information to protect privacy and provides researchers with anonymous identification numbers associated with relevant claims information, including sex, date of birth, medical services received, and prescriptions. Therefore, patient consent is not required to access the NHIRD. This study was approved to fulfill the condition for exemption by the Institutional Review Board (IRB) of China Medical University (CMUH104-REC2-115-CR1). The IRB also specifically waived the consent requirement.

### Sample

We conducted a retrospective cohort of HCV infected patients from the one million patients in representative LHID 2000 sample dataset. The HCV cohort contained adult patients newly diagnosed with HCV infection (ICD-9-CM codes 070.41, 070.44, 070.51, 070.54, and V02.62) from January 1, 2000, to December 31, 2010; the date of first diagnosis was set as the index date. We excluded patients with a history of hepatitis B virus infections (ICD-9-CM codes 070.20, 070.22, 070.30, 070.32, and V02.61) and cataract (ICD-9-CM code 366) in medical records before index date. The comparison cohort was comprised of non-HCV infection patients. To control for the confounding effects of age (every 5-year span), sex, and index year, we constructed a matched variable containing the age at index data and sex for each HCV infection patient from the comparison cohort. All participants were followed from the index date until the date of cataract diagnosis, withdrawal from the NHI program, death, or the end of 2011 (December 31, 2011).

### Comorbidity and medication

All confounding variables were defined according to the diagnosis before the index date in HCV and non-HCV patients. Cirrhosis was defined as three outpatient claims with ICD-9-CM codes 571.2, 571.5, and 571.6. Diabetes was defined as defined as three outpatient claims with ICD-9-CM code 250. The following confounding variables for cataract all defined as three outpatients claims with the corresponding ICD-9 code: hypertension (ICD-9-CM codes 401–405), hyperlipidemia (ICD-9-CM code 272), asthma (ICD-9-CM code 493), chronic obstructive pulmonary disease (COPD; ICD-9-CM codes 491, 492, and 496), coronary artery disease (ICD-9-CM codes 410–414), alcohol-related illness (ICD-9-CM codes 291, 303, 305, 571.0, 571.1, 571.2, 571.3, 790.3, A215, and V11.3), and anxiety (ICD-9-CM code 300.00).

Individuals in the HCV group would visit clinics more regularly than those in the non-HCV group, thus HCV patients may be more likely to be diagnosed with cataract at an earlier stage. To alleviate such surveillance bias, frequency of clinical visits was added as confounding variable.

Regimen of HCV infection, including ribavirin, interferon alpha, and interferon alpha-ribavirin combination therapy were classified for evaluating the effects of the HCV therapy on risk of cataract.

### Statistical analysis

Data on the HCV and non-HCV cohorts involving age, follow-up duration, number and proportion of age group, sex, and comorbidity are presented as means and deviations (SDs). We applied a *t* test for age and follow-up duration and the chi-square test for age group, sex, and comorbidity. The Kaplan–Meier curve analysis was performed to reveal the cumulative incidence of cataract, and the log-rank test was applied to identify the differences between the HCV and non-HCV cohorts. The incidence density rate of cataract was estimated as the number of cataract events identified during the follow-up duration divided by the total follow-up person-years. Univariate and multivariate Cox proportion hazard regression models were used to examine the effect of HCV infection on cataract risk through hazard ratios (HRs) with their respective 95% confidence intervals (CIs). The multivariable models included all statistically significant risk factors identified in the univariable Cox model. SAS 9.4 software (SAS Institute, Cary, NC, USA) was used to analyze all data. All statistical analyses were performed with a two-tailed significance level of 0.05.

## Results

**Fig 1:**
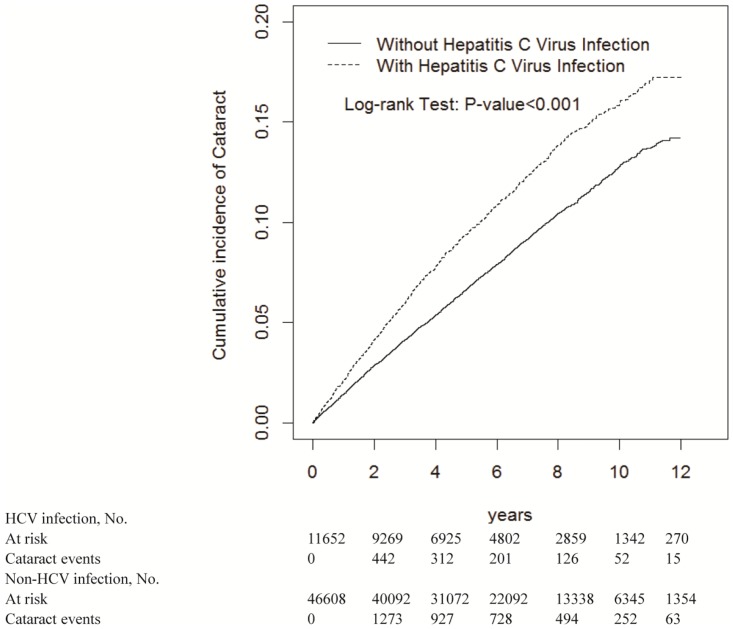
Cummulative incidence comparison of cataract for patients with (dashed line) or without (solid line) HCV infection.

We have enrolled a total of 11,652 HCV patients and 46,608 non-HCV patients. The mean follow-up duration was 5.29 and 5.86 years for the HCV and non-HCV cohorts, respectively. Similar characteristics were recognized in both cohorts which 55.0% were men and 48.3% of participants were aged ≤49 years. The mean ages of the HCV and non-HCV cohorts were 50.5±14.3 and 50.0±14.6 years, respectively. The prevalence of cirrhosis, hypertension, diabetes, hyperlipidemia, asthma, chronic obstructive pulmonary disease, coronary artery disease, alcohol-related illness, and anxiety were significantly in HCV patients, compared with non-HCV patients (*p* <0.001) (Table 1). Kaplan–Meier curve analysis revealed that the HCV cohort exhibited a higher cumulative cataract incidence than the non-HCV cohort did (log-rank test, *p* <0.001; Fig 1).

**Table 1:** Demographic characteristics and comorbidities in cohorts with and without HCV infection.

	HCV infection		
	No	Yes	
Variable	N = 46608	N = 11652	*p*-value
**Age, year**			0.99
≤ 49	22532(48.3)	5633(48.3)	
50–64	16420(35.2)	4105(35.2)	
65+	7656(16.4)	1914(16.4)	
Mean(SD)^†^	50.0(14.6)	50.5(14.3)	0.01
Average number of clinic visits/per year, Mean(SD)	13.5(12.8)	21.0(16.4)	<0.001
**Sex**			0.99
Female	20992(45.0)	5248(45.0)	
Male	25616(55.0)	6404(55.0)	
**Comorbidity**			
Cirrhosis	167(0.36)	1391(11.9)	<0.001
Diabetes	2904(6.23)	1523(13.1)	<0.001
Hypertension	11561(24.8)	3892(33.4)	<0.001
Hyperlipidemia	7175(15.4)	2575(22.1)	<0.001
Asthma	2252(4.83)	856(7.35)	<0.001
COPD	3372(7.23)	1394(12.0)	<0.001
Coronary artery disease	4822(10.4)	1857(15.9)	<0.001
Alcohol-related illness	1322(2.84)	1312(11.3)	<0.001
Anxiety	2269(4.87)	1158(9.94)	<0.001

Chi-Square Test;

^†^: T-Test

There were 1,148 cataract events among 11,752 HCV patients and 3,738 cataract events among 46,608 non-HCV patients. The incidence of cataract was 1.86% (1,148/61,686) in HCV patients. The overall incidence density rate of cataract in the HCV cohort was 1.36 times higher in the non-HCV cohort (1.86 vs. 1.37 per 100 person-y; Table 2). Women had a higher risk of cataract than men (adjusted HR (aHR) = 1.22, 95% CI = 1.15–1.29). After adjusting for age, sex, and comorbidities of diabetes, hypertension, hyperlipidemia, asthma, COPD, coronary artery disease, and anxiety, patients in the HCV cohort were associated with an increased risk of cataract compared with those in the non-HCV cohort [adjusted HR (aHR) = 1.23, 95% CI = 1.14–1.32]. Compared with patients aged ≤49 years, the risk of cataract was 9.23 times higher in those aged 50–64 years (95% CI = 8.29–10.3) and 15.2 times higher in those aged ≥65 years (95% CI = 13.5–17.0). In HCV infection patients, comorbidities independently associated with cataract were diabetes (aHR = 1.41, 95% CI = 1.30–1.52), hypertension (aHR = 1.13, 95% CI = 1.06–1.21), hyperlipidemia (aHR = 1.25, 95% CI = 1.17–1.34), and COPD (aHR = 1.12, 95% CI = 1.03–1.22).

**Table 2:** The incidence and hazard ratio for cataract and cataract-associated risk factor.

Variable	Event	PY	Rate^#^	Crude HR(95% CI)	Adjusted HR^†^ (95% CI)
**HCV infection**					
No	3738	273224	1.37	1.00	1.00
Yes	1148	61686	1.86	1.36(1.27, 1.45)***	1.23(1.14, 1.32)***
**Age, year**					
≤ 49	396	181705	0.22	1.00	1.00
50–64	2686	11684	2.41	11.1(10.0, 12.4)***	9.20(8.27, 10.3)***
65+	1804	41520	4.34	20.2(18.1, 22.6)***	14.8(13.2, 16.6)***
**Sex**					
Female	2653	153042	1.73	1.41(1.34, 1.50)***	1.22(1.15, 1.29)***
Male	2233	181868	1.23	1.00	1.00
**Comorbidity**					
**Cirrhosis**					
No	4728	329724	1.43	1.00	1.00
Yes	158	5186	3.05	2.09(1.79, 2.45)***	1.09(0.92, 1.29)
**Diabetes**					
No	4079	315418	1.29	1.00	1.00
Yes	807	19472	4.14	3.18(2.94, 3.43)***	1.41(1.30, 1.52)***
**Hypertension**					
No	2458	258134	0.95	1.00	1.00
Yes	2428	76776	3.16	3.31(3.13, 3.51)***	1.13(1.06, 1.21)***
**Hyperlipidemia**					
No	3341	285523	1.17	1.00	1.00
Yes	1545	49386	3.13	2.66(2.51, 2.83)***	1.25(1.17, 1.34)***
**Asthma**					
No	4445	320217	1.39	1.00	1.00
Yes	441	14693	3.00	2.14(1.94, 2.37)***	1.01(0.91, 1.13)
**COPD**					
No	4125	312943	1.32	1.00	1.00
Yes	761	21967	3.46	2.61(2.41, 2.82)***	1.12(1.03, 1.22)**
**Coronary artery disease**					
No	3670	302987	1.21	1.00	1.00
Yes	1216	31923	3.81	3.13(2.93, 3.34)***	0.97(0.87, 1.08)
**Alcohol-related illness**					
No	4486	319089	1.41	1.00	1.00
Yes	400	15821	2.53	0.86(0.73, 1.02)	-
**Anxiety**					
No	4743	323774	1.46	1.00	1.00
Yes	143	11136	1.28	1.78(1.60, 1.97)***	0.97(0.87, 1.08)

Rate^#^, incidence rate, per 100 person-years; Crude HR, relative hazard ratio; Adjusted HR^†^: multivariable analysis including age, average number of clinic visits/per year, and comorbidities of diabetes, hypertension, hyperlipidemia, asthma, COPD, coronary artery disease, and anxiety;

** p < 0.01,

*** p<0.001

The incidence of cataract increased with age in both cohorts (Table 3). HCV patients had significant higher risk of cataract across all age categories including aged ≤49 years (aHR = 1.47, 95% CI = 1.18–1.84), 50–64 years (aHR = 1.30, 95% CI = 1.18–1.42), and ≧65 years(aHR = 1.20, 95% CI = 1.06–1.36). The stratification analysis of sex showed that HCV cohort had increasing risks of cataract for both women (aHR = 1.37, 95% CI = 1.24–1.50) and men (aHR = 1.24, 95% CI = 1.11–1.37). The stratification analysis of comorbidities showed that the risk of cataract was aHR = 1.26 (95% CI = 1.16–1.35) for HCV cohort with comorbidities and aHR = 1.45 (95% CI = 1.25–1.69) for HCV cohorts without comorbidities. The interaction analysis of HCV infection and confounding variables showed that significant interaction between HCV infection and diabetes (P for interaction < 0.001), hyperlipidemia (P for interaction = 0.003), and alcohol related disease (P for interaction = 0.003).

**Table 3:** Incidence of cataract by age, sex and comorbidity and Cox model measured hazards ratio for patients with HCV infection compared those without HCV infection.

	HCV infection							
	No			Yes				
Variables	Event	PY	Rate^#^	Event	PY	Rate^#^	Crude HR (95% CI)	Adjusted HR^†^ (95% CI)
**Age, years**								
≤ 49	266	146547	0.18	130	35159	0.37	2.05(1.66, 2.53)***	1.47(1.18, 1.84)***
50–64	2026	91782	2.21	660	19902	3.32	1.52(1.39, 1.66)***	1.30(1.18, 1.42)***
65+	1446	34896	4.14	358	6624	5.40	1.28(1.14, 1.44)***	1.20(1.06, 1.36)**
P for interaction								0.12
**Sex**								
Female	2007	124459	1.61	646	28583	2.26	1.40(1.28, 1.53)***	1.37(1.24, 1.50)***
Male	1731	148765	1.16	502	33103	1.52	1.30(1.18, 1.44)***	1.24(1.11, 1.37)***
P for interaction								0.28
**Comorbidity**^**‡**^								
No	1235	178228	0.69	195	27008	0.72	1.04(0.90, 1.21)	1.45(1.25, 1.69)***
Yes	2503	94996	2.63	953	34678	2.75	1.04(0.97, 1.12)	1.26(1.16, 1.35)***
P for interaction								0.99
**Cirrhosis**								
No	3719	272567	1.36	1009	57157	1.77	1.29(1.21, 1.39)***	1.32(1.23, 1.42)***
Yes	19	657	2.89	139	4529	3.07	1.03(0.64, 1.66)	1.02(0.63, 1.66)
P for interaction								0.41
**Diabetes**								
No	3189	260099	1.23	890	55319	1.61	1.31(1.22, 1.41)***	1.35(1.25, 1.45)***
Yes	549	13125	4.18	258	6367	4.05	0.97(0.84, 1.12)	1.09(0.94, 1.27)
P for interaction								<0.001
**Hypertension**								
No	1935	214096	0.90	523	44038	1.19	1.32(1.20, 1.45)***	1.46(1.32, 1.62)***
Yes	1803	59128	3.05	625	17648	3.54	1.16(1.06, 1.27)**	1.20(1.09, 1.32)***
P for interaction								0.07
**Hyperlipidemia**								
No	2622	236880	1.11	719	48643	1.48	1.33(1.23, 1.45)***	1.31(1.20, 1.43)***
Yes	1116	36344	3.07	429	13043	3.29	1.07(0.96, 1.20)	1.24(1.11, 1.39)***
P for interaction								0.002
**Asthma**								
No	3429	262296	1.31	1016	57921	1.75	1.34(1.25, 1.44)***	1.31(1.22, 1.42)***
Yes	309	10928	2.83	132	3765	3.51	1.23(1.01, 1.51)*	1.28(1.03, 1.58)*
P for interaction								0.47
**COPD**								
No	3213	257267	1.25	912	55676	1.64	1.31(1.22, 1.41)***	1.31(1.21, 1.42)***
Yes	525	15957	3.29	236	6010	3.93	1.19(1.02, 1.39)*	1.26(1.07, 1.47)**
P for interaction								0.28
**Coronary artery disease**								
No	2871	249646	1.15	799	53341	1.50	1.30(1.20, 1.41)***	1.34(1.23, 1.46)***
Yes	867	23578	3.68	349	8345	4.18	1.13(1.00, 1.28)	1.20(1.05, 1.37)**
P for interaction								0.07
**Alcohol-related illness**								
No	3660	267617	1.37	1083	56157	1.93	1.41(1.32, 1.51)***	1.35(1.25, 1.45)***
Yes	78	5607	1.39	65	5529	1.18	0.84(0.61, 1.17)	0.98(0.69, 1.40)
P for interaction								0.003
**Anxiety**								
No	3488	262698	1.33	998	56392	1.77	1.33(1.24, 1.43)***	1.29(1.19, 1.39)***
Yes	250	10527	2.37	150	5294	2.83	1.19(0.97, 1.46)	1.45(1.18, 1.80)***
P for interaction								0.32

Rate^#^, incidence rate, per 100 person-years; Crude HR, relative hazard ratio; Adjusted HR^†^: multivariable analysis including age, average number of clinic visits/per year, and comorbidities of diabetes, hypertension, hyperlipidemia, asthma, COPD, coronary artery disease, and anxiety;

Comorbidity^‡^: Only to have one of comorbidities (including cirrhosis, diabetes, hypertension, hyperlipidemia, asthma, COPD, coronary artery disease, alcohol-related illness and anxiety) classified as the comorbidity group

* p < 0.05,

** p<0.01,

*** p<0.001

Table 4 presents the results of the analysis of the effects of different therapy regimens of HCV infection on the risk of cataract, with the non-HCV cohort as comparison. The onset of cataract from the initiation of HCV treatment was 3.08(SD = 2.66). Compared with non-HCV cohort, associated with risk of cataract was no treatment (aHR = 1.29, 95% CI = 1.20–1.39), interferon-alpha only (aHR = 1.29, 95% CI = 1.20–1.39), and interferon-ribavirin combination (aHR = 1.83, 95% CI = 1.40–2.38). Subgroup analysis showed that HCV patients without cirrhosis receiving interferon-ribavirin combination had significantly higher risk of cataract (aHR = 1.70, 95% CI = 1.28–2.25), compared with non-HCV cohort.

**Table 4:** Incidence, and hazard ratio of cataract between patients with HCV infection with and without treatment.

Variables	N	Event	PY	Rate^#^	Crude HR(95% CI)	Adjusted HR† (95% CI)
Without HCV infection	46608	3738	273224	1.37	1(Reference)	1(Reference)
HCV infection without treatment	11198	1092	58918	1.85	1.35(1.26, 1.45)**	1.29(1.20, 1.39)***
HCV infection with interferon only	27	6	127	4.47	3.44(1.55, 7.66)***	1.29(1.20, 1.39)***
HCV infection with Ribavirin only	10	0	76	0.00	-	-
HCV infection with interferon-Ribavirin combination	454	56	2768	2.02	1.49(1.14, 1.93)**	1.83(1.40, 2.38)***
Without Cirrhosis						
Without HCV infection	46441	3719	272567	1.36	1(Reference)	1(Reference)
HCV infection without treatment	9835	960	54509	1.76	1.29(1.20, 1.38)***	1.30(1.21, 1.40)***
HCV infection with interferon-Ribavirin combination	426	49	2648	1.85	1.36(1.03, 1.81)*	1.70(1.28, 2.25)***

Rate^#^, incidence rate, per 100 person-years; Crude HR, relative hazard ratio; Adjusted HR^†^: multivariable analysis including age, average number of clinic visits/per year, and comorbidities of diabetes, hypertension, hyperlipidemia, asthma, COPD, coronary artery disease, and anxiety;

* p < 0.05,

** p<0.01,

*** p<0.001

Table 5 showed that synergistic effects of HCV with diabetes, hyperlipidemia, and alcohol-related illness on the risk of cataract. Diabetic HCV patients had increasing risk of cataract (aHR = 1.69, 95% CI = 1.48–1.93), compared with non-HCV and non-diabetic patients. HCV patients with accompanying hyperlipidemia had 1.67-fold higher risk of cataract, compared with non-HCV and non-hyperlipidemia patients.

**Table 5:** Cox method estimated hazard ratios of cataract associated HCV infection and comorbidity.

Variables		Crude HR(95% CI)	Adjusted HR^†^ (95% CI)	p-value^#^
**HCV infection**	**Diabetes**			<0.001
No	No	1(Reference)	1(Reference)	
No	Yes	3.39(3.09, 3.71)***	1.44(1.15, 1.34)***	
Yes	No	1.31(1.22, 1.41)***	1.24(1.15, 1.34)***	
Yes	Yes	3.28(2.89, 3.72)***	1.69(1.48, 1.93)***	
**HCV infection**	**Hyperlipidemia**			0.002
No	No	1(Reference)	1(Reference)	
No	Yes	2.76(2.57, 2.96)***	1.39(1.29, 1.50)***	
Yes	No	1.33(1.23, 1.45)***	1.24(1.13, 1.35)***	
Yes	Yes	2.96(2.67, 3.28)***	1.67(1.50, 1.87)***	
**HCV infection**	**Alcohol-related illness**			
No	No	1(Reference)	1(Reference)	0.003
No	Yes	1.00(0.80, 1.25)	1.02(0.81, 1.28)	
Yes	No	1.41(1.32, 1.51)***	1.24(1.15, 1.33)***	
Yes	Yes	0.85(0.66, 1.08)	1.04(0.81, 1.330	

Crude HR, relative hazard ratio;

^†^ Model was adjusted for age, average number of clinic visits/per year, and comorbidities of diabetes, hypertension, hyperlipidemia, asthma, COPD, coronary artery disease, and anxiety;

^#^p-value for interaction;

*** p < 0.001

## Discussion

In this population-based cohort study, we observed that HCV infection, even in the absence of cirrhosis, was associated with an increased risk of cataract. HCV-infected patients receiving interferon–ribavirin therapy had a 1.83 times higher risk of cataract compared with those without HCV infection and not receiving interferon–ribavirin therapy. This suggested that all patients with HCV, regardless of receiving HCV treatment, should be closely monitored for subsequent cataract development.

Even after adjusting for major confounding variables such as age and diabetes, HCV infection remained independently associated with cataract. The effects of HCV on ocular manifestations have been seldom studied. We hypothesized that the pathogenic mechanism connecting HCV infection and cataract is oxidative stress. Evidence has increasingly indicated that HCV infection-induced oxidative stress is systemic and not limited to the liver [18–21]. HCV-infected patients have high insulin resistance and oxidative stress, which increases their risk of coronary artery disease [22], chronic kidney disease [23], glucose metabolism derangements, and diabetes [24]. Similarly, HCV may induce high oxidative stress within the lens, initiating a cascade of free radial formation, oxidized material accumulation, and ultimately, cataract development. Because we have adjusted our data for diabetes, a major risk factor for cataract and a recognized sequela of HCV infection, our results clearly demonstrated an independent association between HCV infection and cataract.

In this study, we observed a positive association between pegylated interferon and ribavirin therapy for HCV infection and risk of cataract. Our data showed that HCV patients receiving interferon alpha-only regimen had 1.29-fold higher risk of cataract, interferon alpha-ribavirin regimen had 1.83-fold higher risk of cataract, compared with non-HCV patients. Thus, the regimen combining Interferon alpha and ribavirin would have addictive and synergistic effects on the risk of developing cataract. Few case reports and studies with small sample sizes have reported similar findings [25, 26]. This current large-scale cohort study clearly demonstrated that pegylated interferon and ribavirin therapy for HCV infection is associated with a 1.83 times higher risk of cataract. Interferon-associated retinopathy has been described in the literature [27, 28], interferon alfa–ribavirin-associated cataract has seldom been mentioned. There are several possible explanations accounting for the association between HCV therapy and cataract. One supposed pathway would be direct effects of interferon/ribavirin treatment or to HCV itself. It has been reported that about 0.5% patients taking ribavirin have cataract, with the incidence being up to 55.36% during the initial 6 months of ribavirin use, and the most co-used medication in these cataract patients was interferon alfa-2a [29]. The sample size of our HCV patients receiving ribavirin only was too small to determine whether ribavirin would be direct related to the risk of cataract. Further prospective randomized control study would be needed to classify the association between ribavirin and cataract.

Our HCV cohort had significantly higher prevalence of comorbidities compared with non-HCV cohort. Our interaction analysis showed that HCV interacted with diabetes, hyperlipidemia, and alcohol-related illness on the increasing risk of cataract. HCV patients had diabetes had highest risk of cataract, among those with or without HCV or diabetes. Our data demonstrated that clinical relevance of HCV infection is not equal to those of the other confounding factors of high oxidative stress, including diabetes and aging.

The other possible explanations would be competing risk between HCV-related mortality and cataract. It would be reasonable to speculate that systemic oxidative stress would be positively associated with the degree of HCV infection. However, in our stratified analysis, there was no significant difference in developing cataract between cirrhosis and non-cirrhosis. Thus, we suppose that there are competing risk between cataract and HCV- related morbidity and mortality in those HCV decompensation patients. On the other hand, current guideline indicated therapy should be considered for all compensated treatment—naïve HCV patients [30]. Thus, possible bias resulting from competing risk would be minimized in those HCV patients receiving interferon–ribavirin therapy since those who receiving therapy all had compensated liver function. However, possible selection bias would exist since viral load of HCV, genotype of HCV, status of liver reserve, and mounted immune response to therapy, these possible predisposing factors of cataract, of whom receiving interferon/ribavirin would be different at baseline from those HCV patients not receiving interferon/ribavirin. Further large scale prospective study would be required to investigate the association between HCV treatment and cataract.

This study has several limitations. First, information regarding the cataract subtype and family history of each cataract patient was unavailable. Second, details regarding smoking habit, vitamin supplement use, ultraviolet exposure, and sun protection were not available from the ICD-9-CM codes. Third, because the NHIRD encrypts patient personal information to protect privacy, no records regarding HCV genotype, viral load of HCV, serological response to HCV treatment, and severity of cataract were available; therefore, a direct and substantial dose–response relationship between HCV infection and cataract could not be established. Nevertheless, we controlled for possible confounding variables such as frequency of clinical visits, diabetes, coronary artery disease, hypertension, hyperlipidemia, COPD, asthma, and alcohol-related illness; thereafter, HCV infection was found to be independently associated with risk of cataract. Finally, treatment outcome, i.e. response to HCV therapy was unavailable in NHIRD. However, lack of treatment outcome of HCV therapy would not have effects on our conclusion that HCV infection is associated with an increased risk of cataract especially undergoing interferon–ribavirin therapy.

In conclusion, the current nationwide cohort study revealed that HCV-infected patients have an increased risk of cataract. Furthermore, HCV-infected patients receiving interferon–ribavirin therapy have a 1.83 times higher prevalence of cataract than those without HCV infection. Considering the surgical curability of cataract and serious HCV infection-related morbidity, we do not discourage the use of anti-HCV therapy for HCV-infected patients. Instead, we recommend routine screening of these HCV patients for ocular problems, especially those received interferon alpha–ribavirin therapy.

## Supporting information

S1 STROBE Checklist Checklist of items that should be included in reports of observational studies. (DOC)

## Abbreviations

CI: confidence interval
HCV: Hepatitis C virus
HR: hazard ratio
LHID2000: Longitudinal Health Insurance Database 2000
SD: standard deviation
